# Supplementary material for: Polypolish: Short-read polishing of long-read bacterial genome assemblies
Source: PLoS Comput Biol. 2022 Jan 24;18(1):e1009802. doi: 10.1371/journal.pcbi.1009802 (PMC8812927; doi:10.1371/journal.pcbi.1009802)
Supplement: S1 Fig — (PDF) [file pcbi.1009802.s001.pdf]

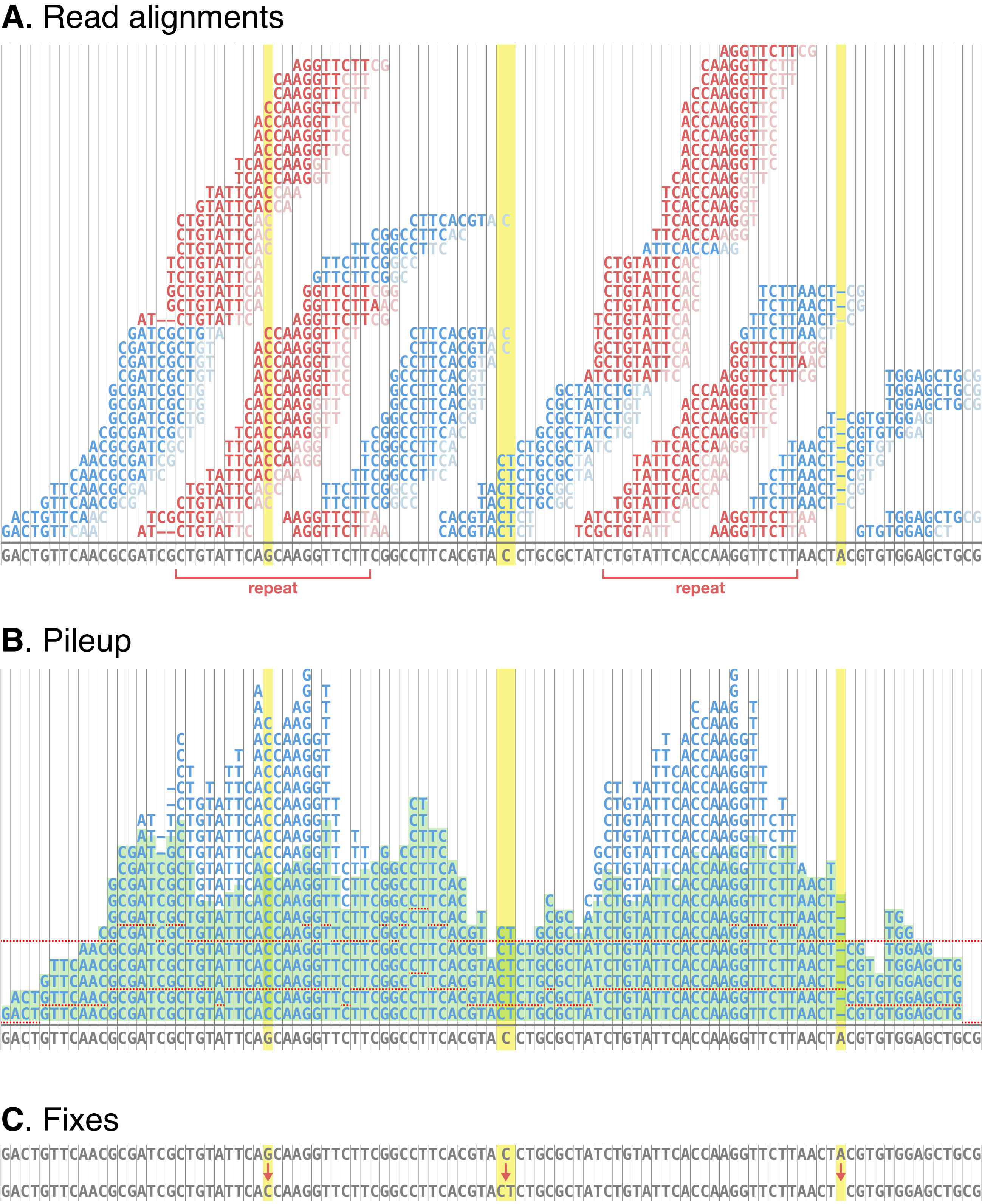

Figure S1: illustrated steps of the Polypolish algorithm. Assembly positions containing an error are highlighted in yellow.

A: short-read alignments to the assembly sequence. Reads which aligned to multiple positions are coloured red, and reads which aligned to only one position are coloured blue. Trimmed bases from the end of each alignment are shown in dimmed type.

B: pileup generated from alignments. Calculated read depth is shown with green shading. The valid (upper) and invalid (lower) thresholds are shown as dotted red lines.

C: fixes to the assembly sequence. Polypolish makes changes at any position where there is one and only one valid sequence, that sequence differs from the assembly base, and all other sequences are invalid.
