# Supplementary material for: Polypolish: Short-read polishing of long-read bacterial genome assemblies
Source: PLoS Comput Biol. 2022 Jan 24;18(1):e1009802. doi: 10.1371/journal.pcbi.1009802 (PMC8812927; doi:10.1371/journal.pcbi.1009802)
Supplement: S2 Fig — (PDF) [file pcbi.1009802.s002.pdf]

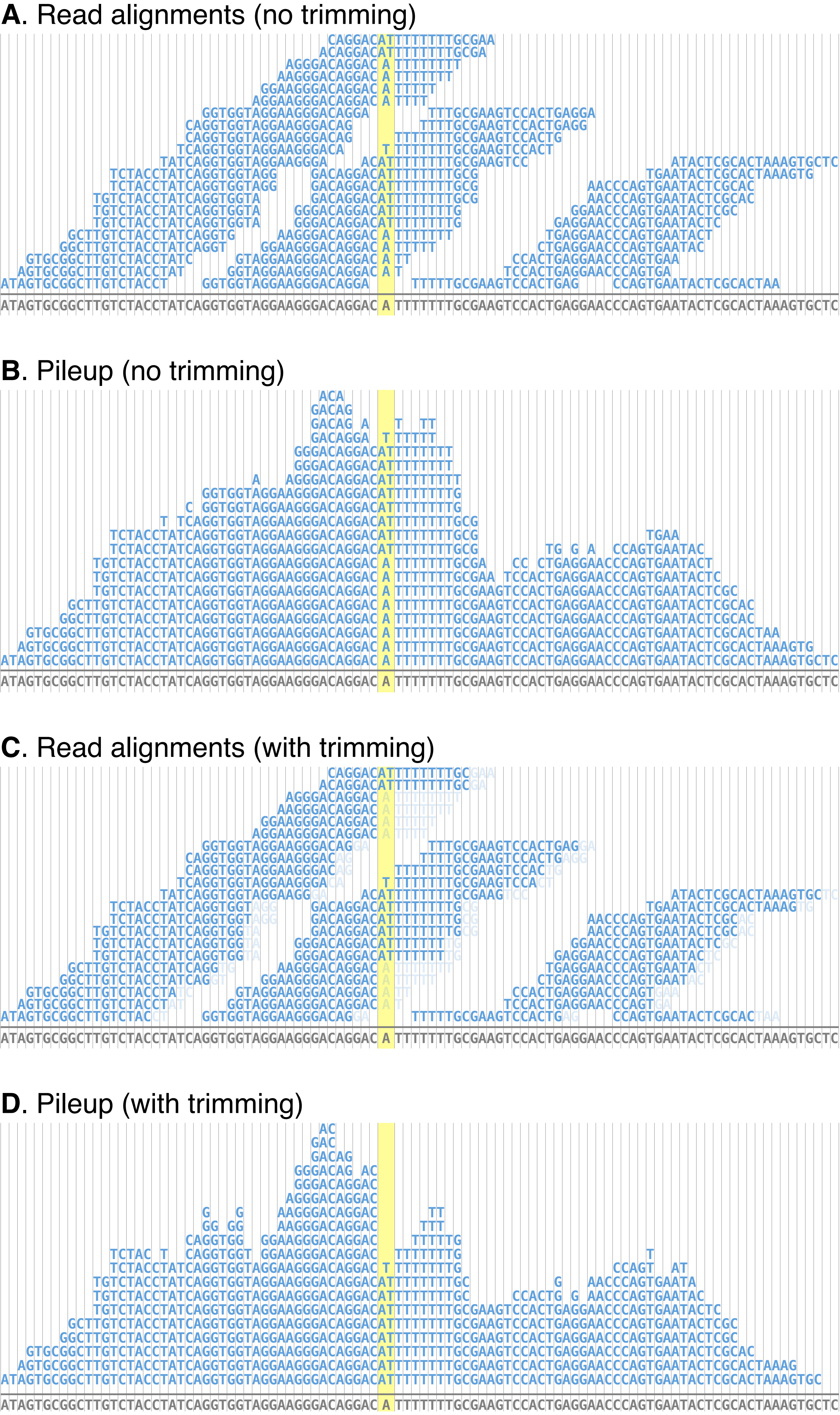


**Figure S2**: alignment trimming in the Polypolish algorithm. The assembly sequence contains a homopolymer-length error, the position of which is highlighted in yellow.

**A:** short-read alignments to the assembly sequence without any alignment trimming.

**B:** pileup generated from the untrimmed alignments. The error-containing position contains multiple instances of both 'AT' (the correct call) and 'A' (an incorrect call).

**C:** short-read alignments to the assembly sequence where bases have been trimmed from the end of each alignment: the last base has been trimmed (however many times it occurs) and one additional base. Trimmed bases are shown in dimmed type.

**D:** pileup generated from the trimmed alignments. The error-containing position no longer contains any instances of the incorrect call 'A'.
