# Supplementary material for: Polypolish: Short-read polishing of long-read bacterial genome assemblies
Source: PLoS Comput Biol. 2022 Jan 24;18(1):e1009802. doi: 10.1371/journal.pcbi.1009802 (PMC8812927; doi:10.1371/journal.pcbi.1009802)
Supplement: S3 Fig — (PDF) [file pcbi.1009802.s003.pdf]

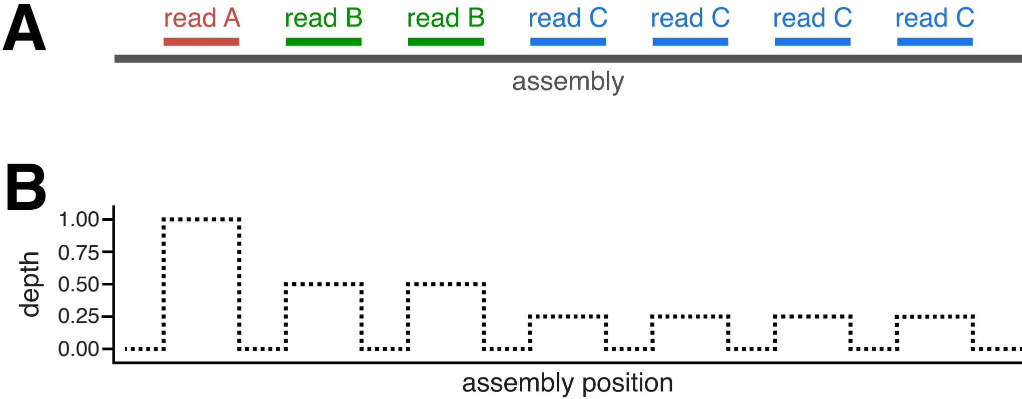

Figure S3: Fractional read depth in the Polypolish algorithm.

A: hypothetical read alignments for three 100 bp reads to a 1200 bp assembly. Read A aligned to a single location, read B to two locations and read C to four locations.

B: Polypolish read depths resulting from the alignments. Each alignment contributes $1/n$ depth, where n is the total number of alignments for that read. This results in a mean depth of 0.25 for the entire assembly, the same value which would be obtained if each read only had a single alignment.
