# Supplementary material for: Polypolish: Short-read polishing of long-read bacterial genome assemblies
Source: PLoS Comput Biol. 2022 Jan 24;18(1):e1009802. doi: 10.1371/journal.pcbi.1009802 (PMC8812927; doi:10.1371/journal.pcbi.1009802)
Supplement: S4 Fig — (PDF) [file pcbi.1009802.s004.pdf]

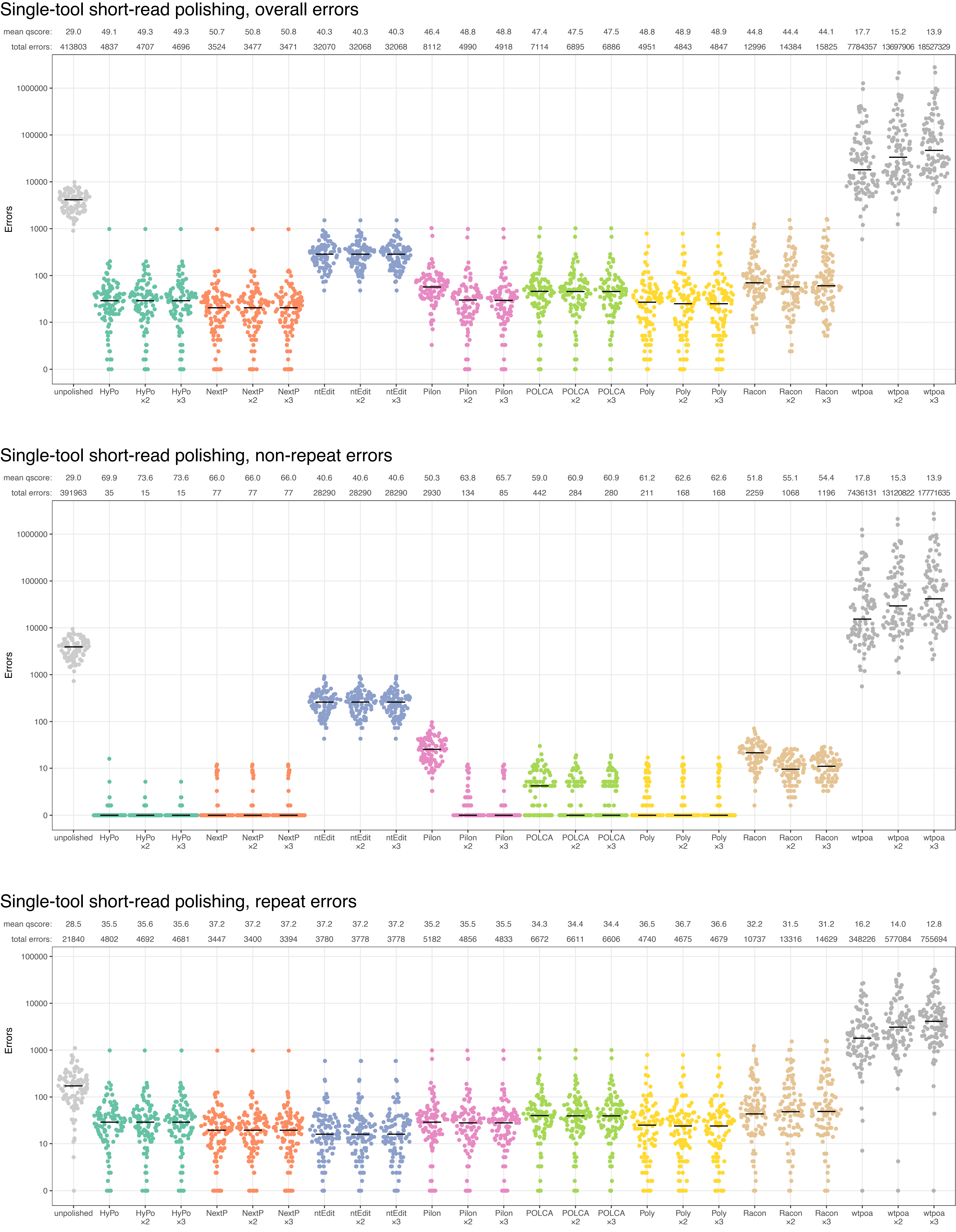

Figure S4: short-read polishing tool benchmarking results using 100 genomes with simulated Illumina reads. This figure shows the same results as Figure 2A but with additional detail: all polishing rounds are shown, wtpoa is included, and results are broken down for non-repeat regions and repeat regions of the genomes. Mean qscores and error totals are shown at the top of the plot, and the horizontal lines indicate median error rates for each polisher.
