# Supplementary material for: Polypolish: Short-read polishing of long-read bacterial genome assemblies
Source: PLoS Comput Biol. 2022 Jan 24;18(1):e1009802. doi: 10.1371/journal.pcbi.1009802 (PMC8812927; doi:10.1371/journal.pcbi.1009802)
Supplement: S5 Fig — (PDF) [file pcbi.1009802.s005.pdf]

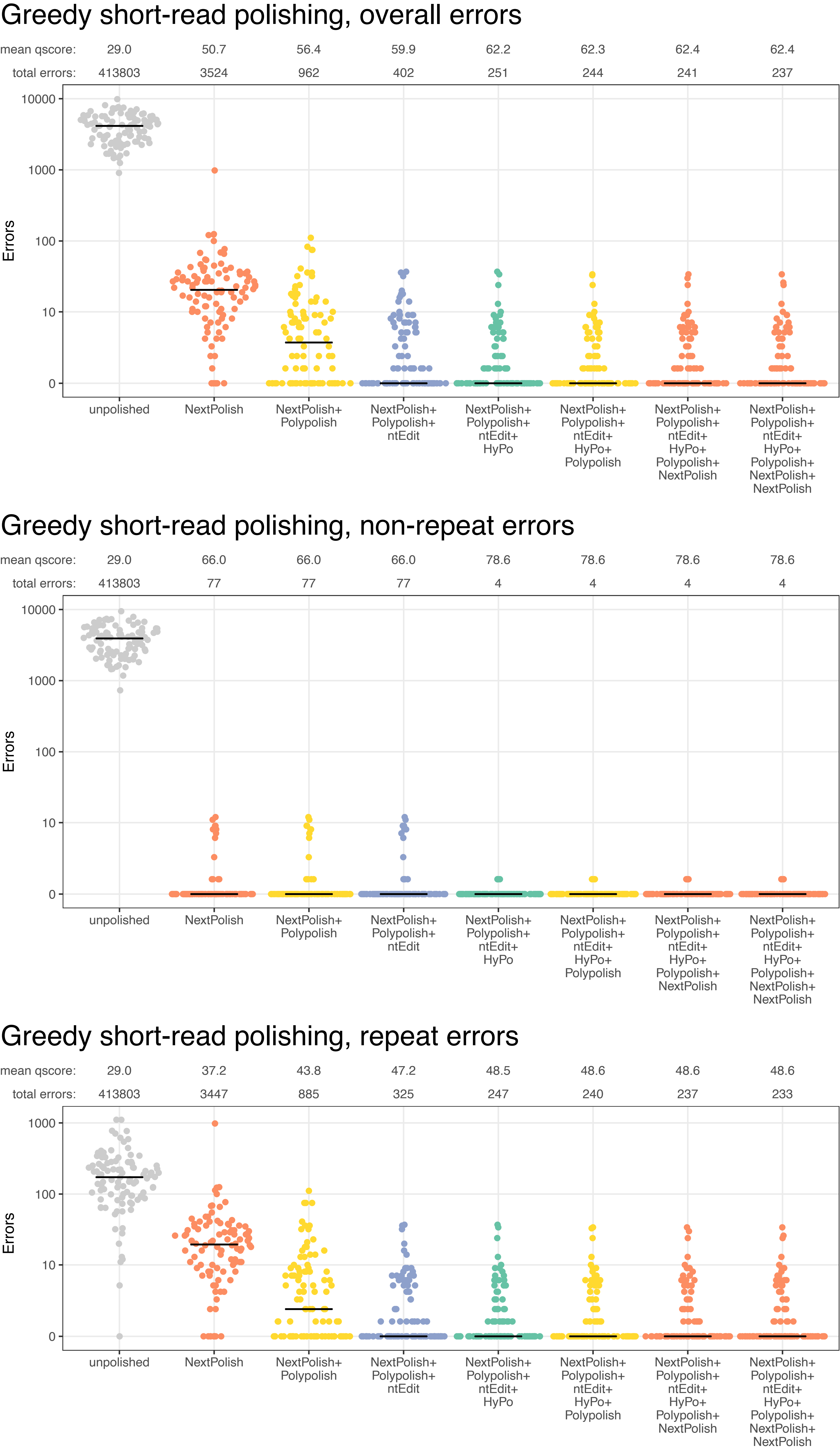

Figure S5: short-read polishing tool benchmarking results using 100 genomes with simulated Illumina reads. Per-genome error rates are shown for the best-performing polisher for each round of the greedy combination test (see Figure 2B), and results are broken down for non-repeat regions and repeat regions of the genomes. Mean qscores and error totals are shown at the top of the plot, and the horizontal lines indicate median error rates for each polisher.
