# Supplementary material for: Polypolish: Short-read polishing of long-read bacterial genome assemblies
Source: PLoS Comput Biol. 2022 Jan 24;18(1):e1009802. doi: 10.1371/journal.pcbi.1009802 (PMC8812927; doi:10.1371/journal.pcbi.1009802)
Supplement: S6 Fig — (PDF) [file pcbi.1009802.s006.pdf]

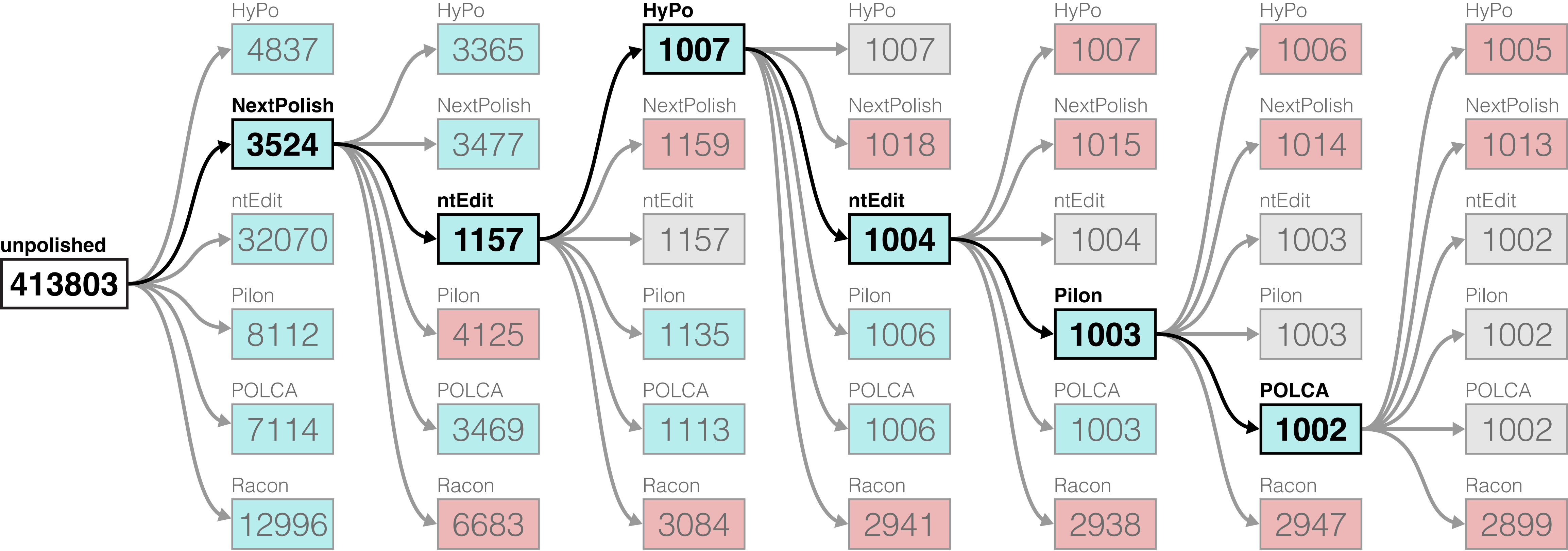

Figure S6: simulated-read greedy polishing error totals, following the same method and format as Figure 2B but excluding Polypolish. The tie in round 5 (between Pilon and POLCA) was broken using ALE score totals (larger for Pilon).
