# Supplementary material for: Polypolish: Short-read polishing of long-read bacterial genome assemblies
Source: PLoS Comput Biol. 2022 Jan 24;18(1):e1009802. doi: 10.1371/journal.pcbi.1009802 (PMC8812927; doi:10.1371/journal.pcbi.1009802)
Supplement: S8 Fig — (PDF) [file pcbi.1009802.s008.pdf]

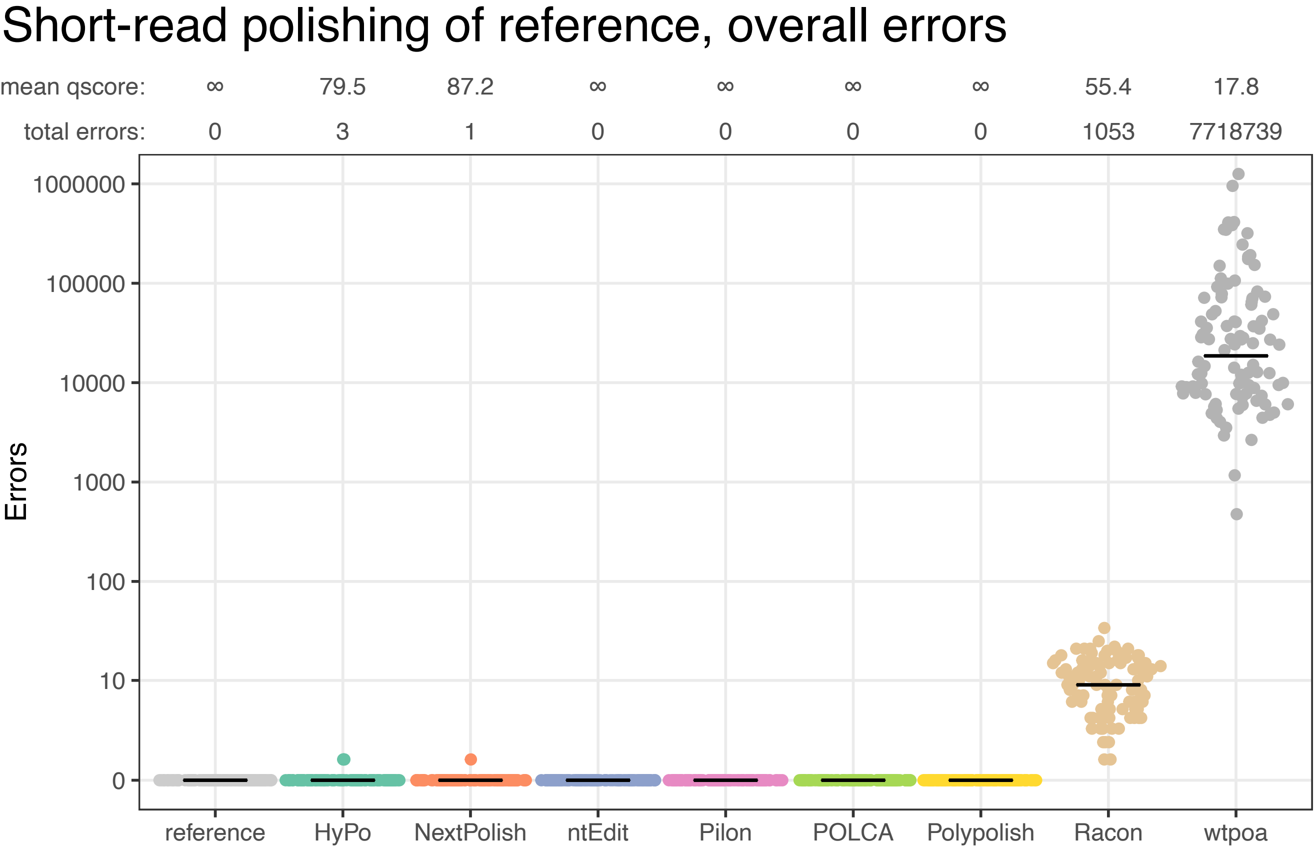

Figure S8: short-read polishing tool benchmarking results using 100 genomes with simulated Illumina reads, using the error-free reference sequence as input. Per-genome error rates are shown after one round of polishing with a single tool. Mean qscores and error totals are shown at the top of the plot, and the horizontal lines indicate median error rates for each polisher.
