# Supplementary material for: Polypolish: Short-read polishing of long-read bacterial genome assemblies
Source: PLoS Comput Biol. 2022 Jan 24;18(1):e1009802. doi: 10.1371/journal.pcbi.1009802 (PMC8812927; doi:10.1371/journal.pcbi.1009802)
Supplement: S9 Fig — (PDF) [file pcbi.1009802.s009.pdf]

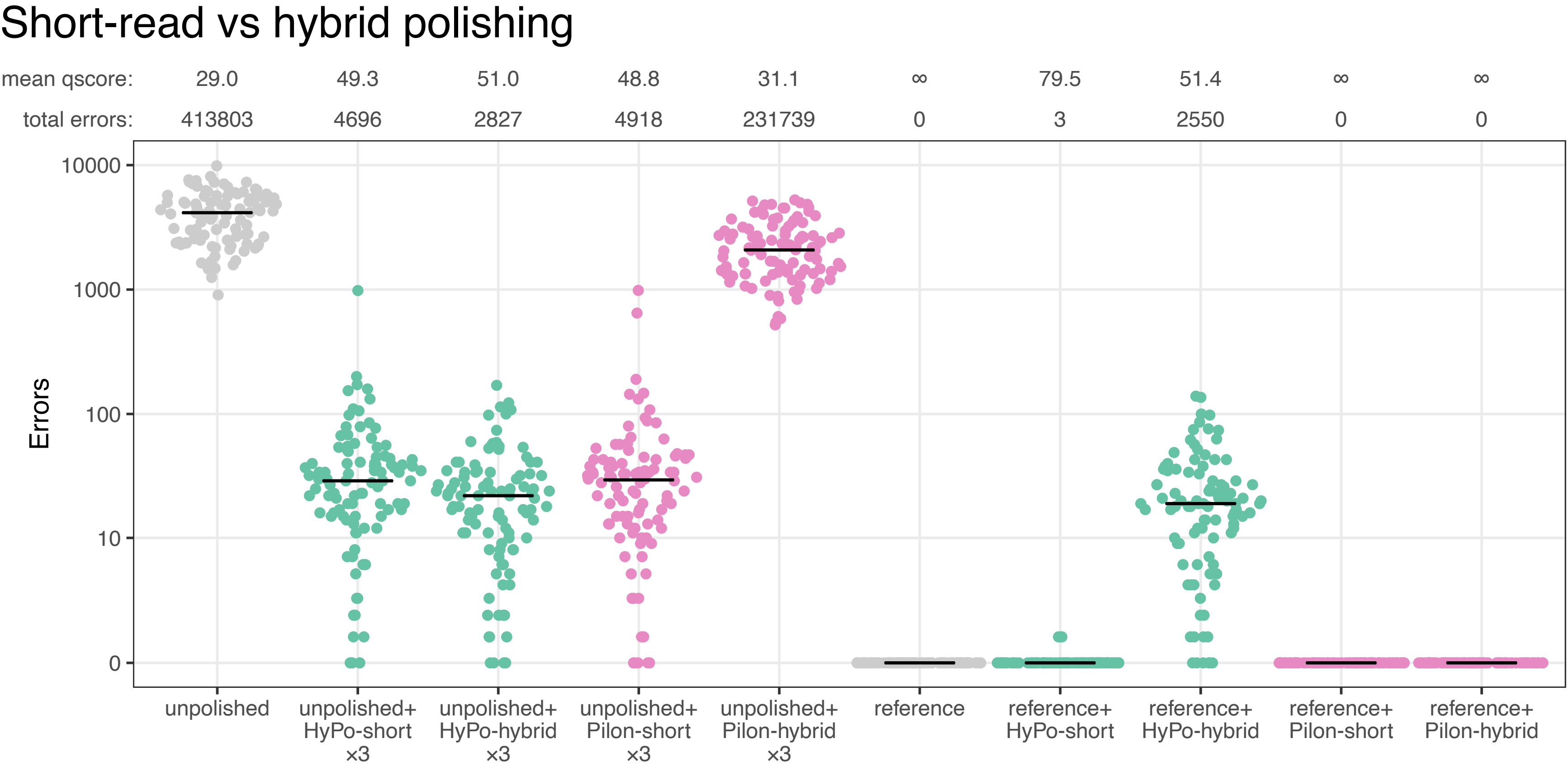

Figure S9: short-read vs hybrid polishing benchmarking results using 100 genomes with simulated Illumina reads. Using the unpolished error-containing sequence as input, per-genome error rates are shown after three rounds of polishing with a single tool. Using the error-free reference sequence as input, per-genome error rates are shown after one round of polishing with a single tool. Mean qscores and error totals are shown at the top of the plot, and the horizontal lines indicate median error rates for each polisher.
