# Supplementary material for: Polypolish: Short-read polishing of long-read bacterial genome assemblies
Source: PLoS Comput Biol. 2022 Jan 24;18(1):e1009802. doi: 10.1371/journal.pcbi.1009802 (PMC8812927; doi:10.1371/journal.pcbi.1009802)
Supplement: S10 Fig — (PDF) [file pcbi.1009802.s010.pdf]

Per-genome ALE score rank vs identity rank

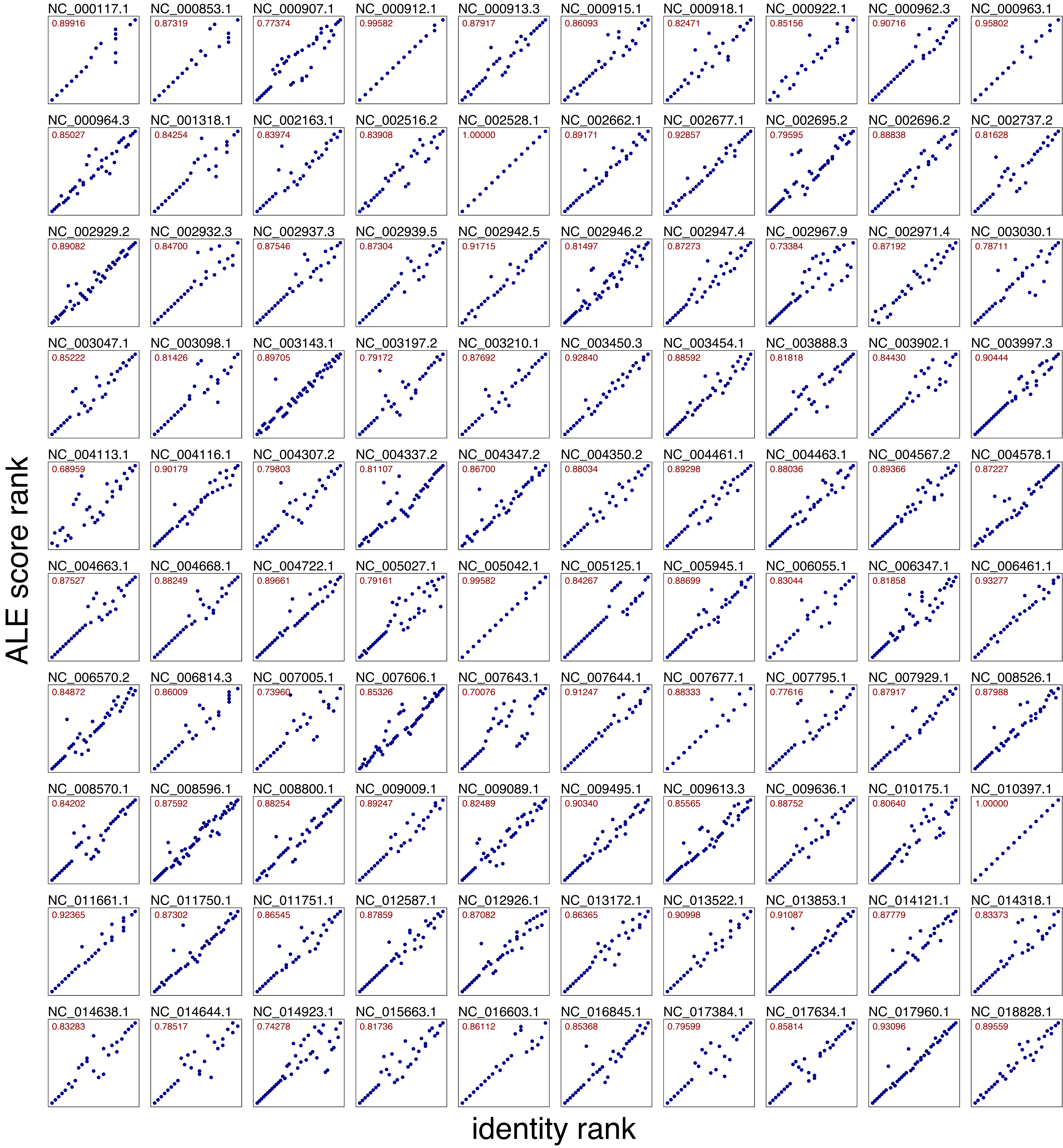

Figure S10: ALE score rank vs identity rank correlations for each of the 100 simulated-read genomes. Each plot contains all unique assembly sequences available for that genome. ALE scores were produced by ALE using short-read alignments to the assembly. Identity was determined using global alignment of the assembly to the original reference sequence. The number in each plot is the Kendall rank correlation coefficient (tau). Of all predictors of assembly quality tested, ALE score had the highest mean tau value (Table S4).
