# Supplementary material for: Polypolish: Short-read polishing of long-read bacterial genome assemblies
Source: PLoS Comput Biol. 2022 Jan 24;18(1):e1009802. doi: 10.1371/journal.pcbi.1009802 (PMC8812927; doi:10.1371/journal.pcbi.1009802)
Supplement: S11 Fig — (PDF) [file pcbi.1009802.s011.pdf]

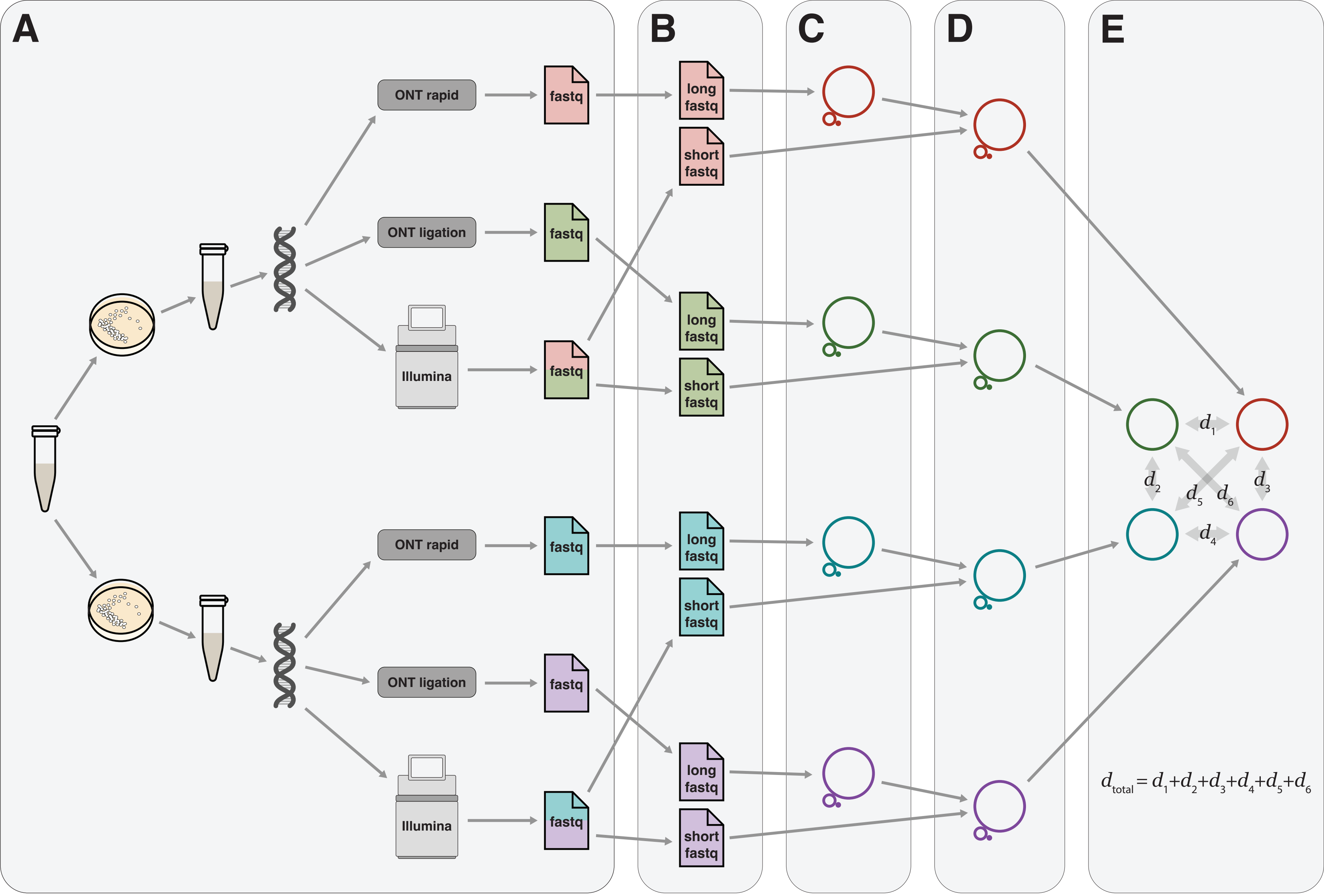


**Figure S11**: sequencing, assembly and distance methods for the real-read tests.

**A:** each genome was sequenced using three different methods (ONT rapid, ONT ligation and Illumina) from a single DNA extraction. A second DNA extraction was then used to sequence each isolate again with each method, yielding a total of six read sets (four ONT and two Illumina) for each isolate.

**B:** Illumina read sets were evenly split into two and combined with ONT read sets to produce four independent hybrid read sets.

**C:** the long-read component of each hybrid read set was used to produce Trycycler assemblies (long-read polished with Medaka).

**D:** short-read polishing was performed using the long-read assemblies from the previous step with the short-read component of each hybrid read set. This polishing was performed many times using each of the tools, as necessary to carry out both the single-tool and greedy-combination tests.

**E:** all pairwise global alignments were performed on the chromosome sequences from each assembly. The edit distance from each alignment was calculated, and the sum of all pairwise edit distances ($d_{total}$) was used as a metric of polisher accuracy.
