# Supplementary material for: Polypolish: Short-read polishing of long-read bacterial genome assemblies
Source: PLoS Comput Biol. 2022 Jan 24;18(1):e1009802. doi: 10.1371/journal.pcbi.1009802 (PMC8812927; doi:10.1371/journal.pcbi.1009802)
Supplement: S12 Fig — (PDF) [file pcbi.1009802.s012.pdf]

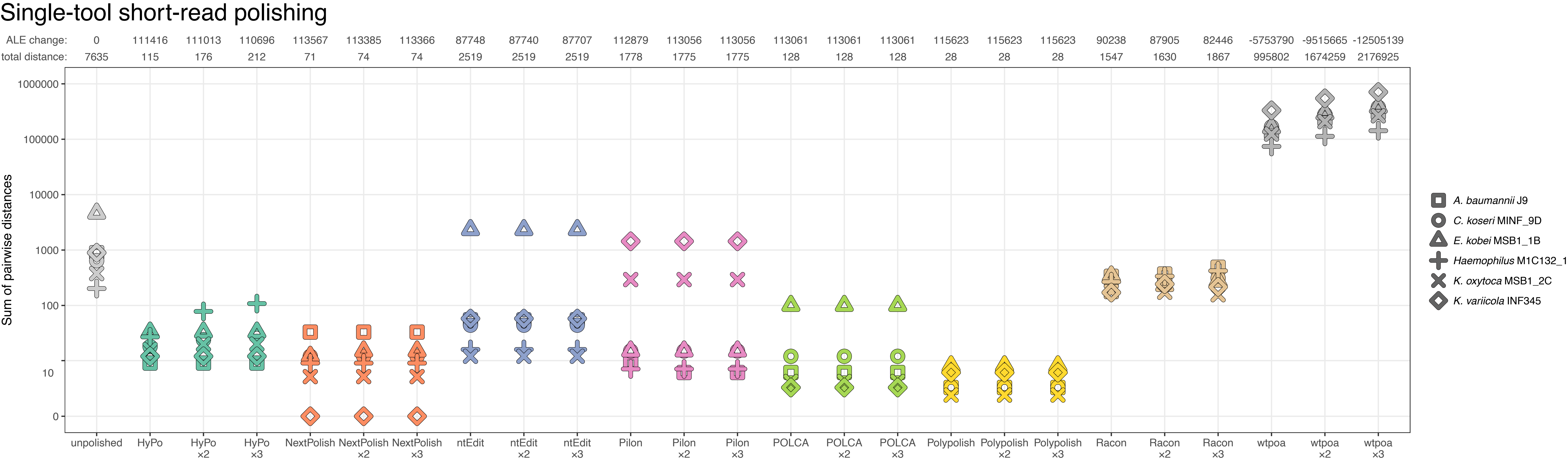

Figure S12: short-read polishing tool benchmarking results using six clusters of genomes with real Illumina reads. This figure shows the same results as Figure 3A but with additional detail: all polishing rounds are shown and wtpoa is included. Distance totals (lower is better) and mean ALE scores relative to the unpolished genomes (higher is better) are shown at the top of the plot.
