# Supplementary material for: Polypolish: Short-read polishing of long-read bacterial genome assemblies
Source: PLoS Comput Biol. 2022 Jan 24;18(1):e1009802. doi: 10.1371/journal.pcbi.1009802 (PMC8812927; doi:10.1371/journal.pcbi.1009802)
Supplement: S13 Fig — (PDF) [file pcbi.1009802.s013.pdf]

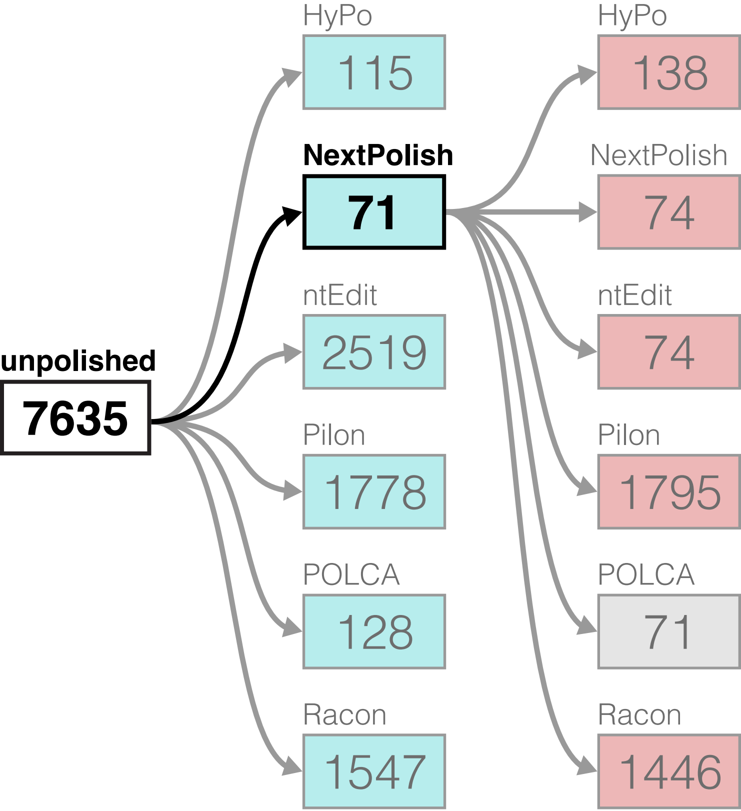

Figure S13: real-read greedy polishing error totals, following the same method and format as Figure 3B but excluding Polypolish.
