# Supplementary material for: Polypolish: Short-read polishing of long-read bacterial genome assemblies
Source: PLoS Comput Biol. 2022 Jan 24;18(1):e1009802. doi: 10.1371/journal.pcbi.1009802 (PMC8812927; doi:10.1371/journal.pcbi.1009802)
Supplement: S14 Fig — (PDF) [file pcbi.1009802.s014.pdf]

A. *K. oxytoca* MSB1_2C Polypolish+POLCA (3,609,000 bp to 3,610,444 bp)

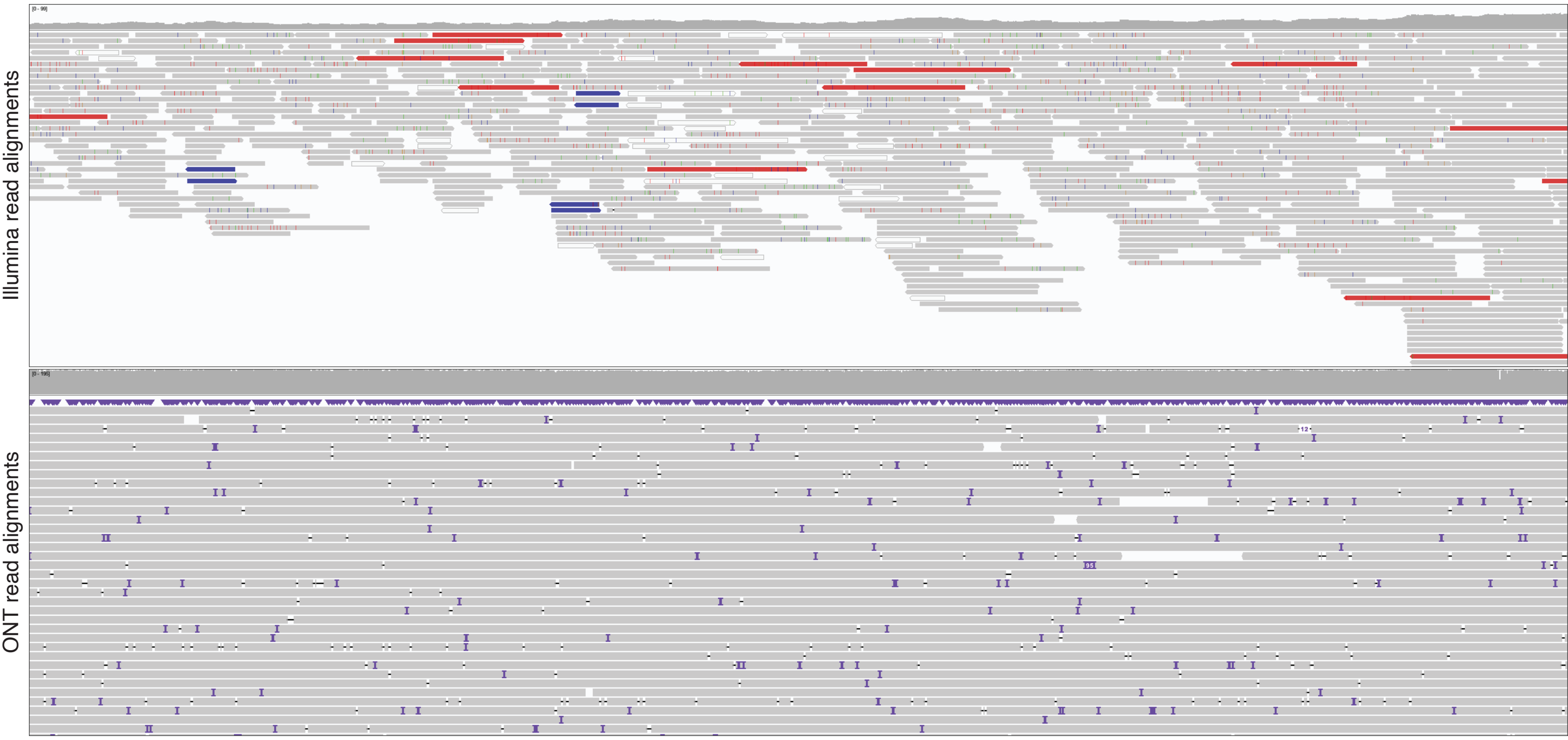

B. *K. oxytoca* MSB1_2C Polypolish+POLCA+Pilon (3,609,000 bp to 3,610,300 bp)

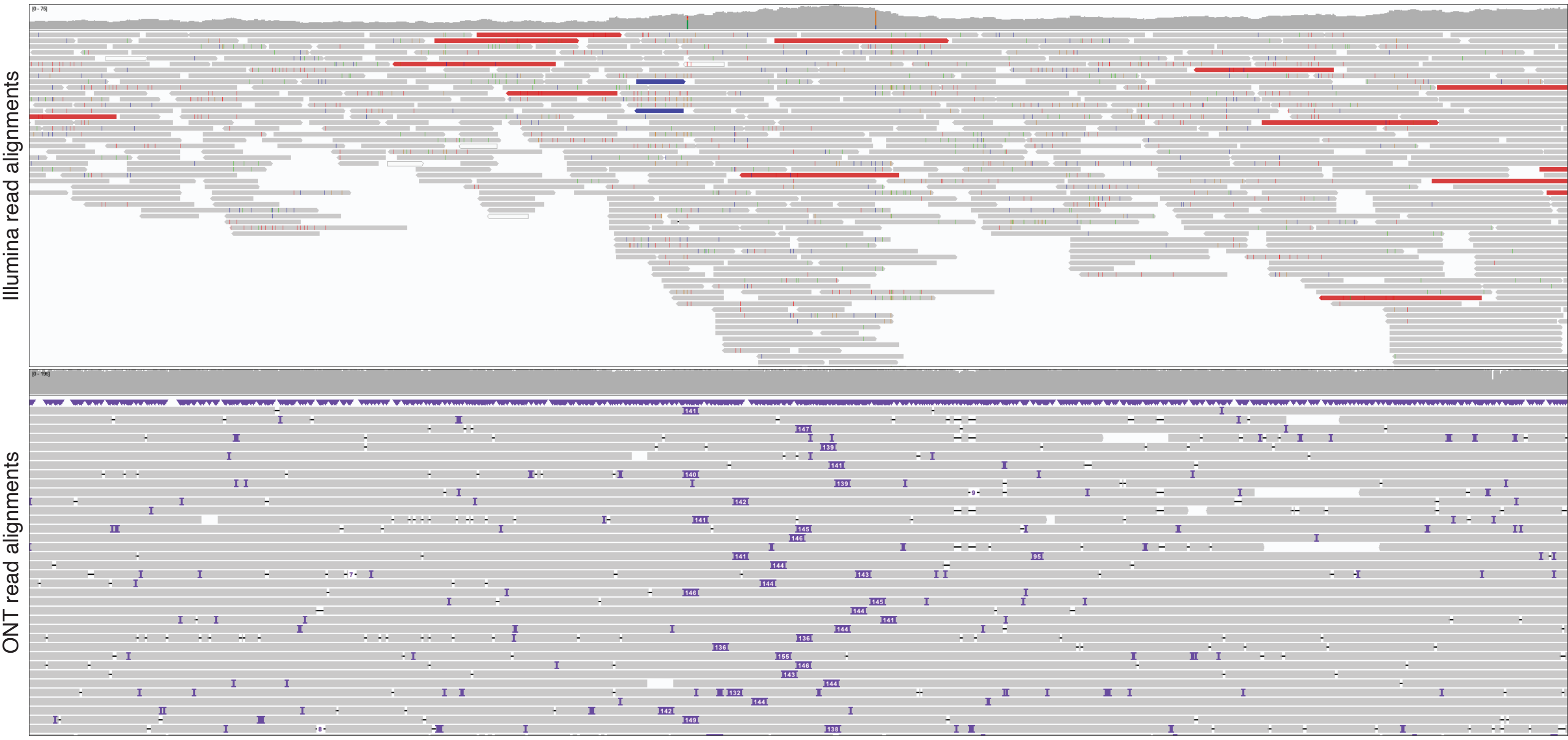

Figure S14: Illumina and ONT read alignments to assemblies of the *K. oxytoca* MSB1_2C read-set-A genome, as visualised by the Integrated Genomics Viewer (IGV), before and after one round of Pilon polishing. The alignments are shown for a repetitive region of interest where Pilon polishing resulted in the deletion of 144 bp (the only change made by Pilon in this case).

A: before Pilon polishing, the Illumina read alignments have low depth across the region of interest, and the ONT read alignments show few large deletions. The ALE score for this assembly (based on Illumina read alignments) was -27262808.

B: after Pilon polishing, the Illumina read alignments are deeper, but most ONT read alignments which span the region contain a large insertion approximately 144 bp in size, showing that the change made by Pilon was in error. The ALE score for this assembly (based on Illumina read alignments) was -27261633, which is larger than the pre-Pilon ALE score by 1175. ALE score is therefore not correlated with assembly accuracy in this case.
