# Supplementary material for: Polypolish: Short-read polishing of long-read bacterial genome assemblies
Source: PLoS Comput Biol. 2022 Jan 24;18(1):e1009802. doi: 10.1371/journal.pcbi.1009802 (PMC8812927; doi:10.1371/journal.pcbi.1009802)
Supplement: S7 Fig — (PDF) [file pcbi.1009802.s007.pdf]

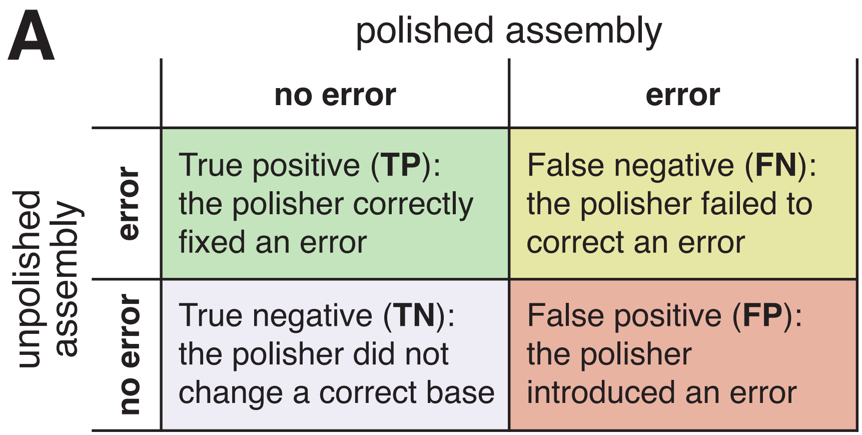

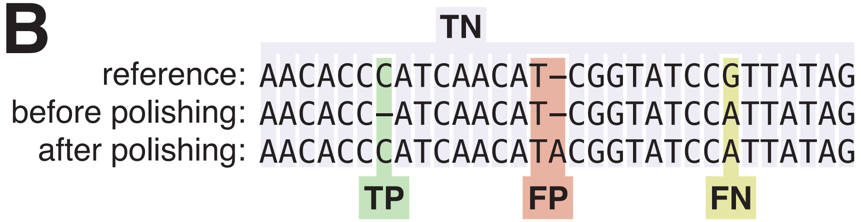

C

Polisher	TP	TN	FP	FN
HyPo	406804	366150176	51	4766
NextPolish	408177	366150103	102	3415
ntEdit	376561	366149853	371	35012
Pilon	406393	366147448	2760	5196
POLCA	404541	366150151	37	7068
Polypolish	406627	366150226	2	4942
Racon	403936	366145001	5161	7699
wtpoa	397472	357964313	8183772	16240

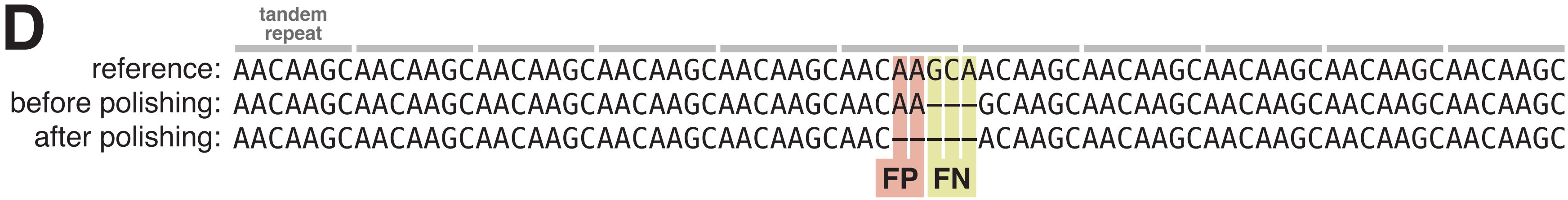

Figure S7: confusion matrices for the simulated-read tests.

A: Definitions of true positive (TP), false positive (FP), true negative (TN) and false negative (FN) classifications in the context of assembly polishing.

B: Labelled sample sequence. A result (TP, TN, FP or FN) is given to each base in the reference, which can include multiple assembly bases in the case of insertion errors.

C: TP, TN, FP and FN totals across all 100 simulated-read test genomes for each short-read polishing tool. These values compare unpolished sequences to the result of a single round of polishing. Polypolish performed best on TN and FP metrics (i.e. at error-free positions of the assembly) and NextPolish performed best on TP and FN metrics (i.e. at error-containing positions of the assembly).

D: The only instance of false positives in Polypolish assemblies occurred in the NC_002967.9 genome. The unpolished assembly contained a 3-bp deletion in a 7-bp tandem repeat, and Polypolish erroneously changed this into a 5-bp deletion, which (on a per-reference-base basis) created two false positives and three false negatives.
